# Cycloaddition of 1,3-Butadiynes: Efficient Synthesis of Carbo- and Heterocycles

**DOI:** 10.3390/molecules190913788

**Published:** 2014-09-03

**Authors:** Tauqir A. Nizami, Ruimao Hua

**Affiliations:** Department of Chemistry, Tsinghua University, Beijing 100084, China; E-Mail: tauqirnizami@gmail.com

**Keywords:** 1,3-butadiynes, cycloaddition, cabocycles, heterocycles

## Abstract

Cycloaddition reactions of alkynes are elegant, atom-efficient transformations for the synthesis of carbo- and heterocycles, mostly aromatic, involving the construction of challenging skeletons of complex molecules. Therefore significant efforts have recently been devoted to the development of novel methodologies, efficient strategies and different catalytic systems to broaden the scope of these reactions. We summarize in this review the recent advances in the cycloaddition reactions of 1,3-butadiynes to provide facile and reliable approaches to various functionalized carbo- and heterocycles.

## 1. Introduction

The carbon-carbon triple bond of alkynes is one of the basic groups in organic synthesis because of their rigidity, electronic properties, and versatile reactivity [1]. In the past decades, acetylene chemistry has experienced a renaissance, due to not only its occurrence in molecules in the frontiers of organic chemistry such as biochemistry or material sciences, but also as building blocks or versatile intermediates for the synthesis of a vast array of chemicals [2].

Among the alkynes, diynes are the most interesting ones since they can be used as key precursors to convey molecular complexity [3]. Diynes are also important tools and structural elements in material sciences, chemical biology [1], photonic materials [4], oligomers and polymers [5,6,7]. Particularly 1,3-butadiynes are versatile building blocks in the construction of linearly π-conjugated acetylenic oligomers and polymers [8,9,10,11], natural products [12], electronic and optical materials [13], and molecular recognition systems [14,15]. Thus numerous synthetic approaches have been developed for the synthesis of 1,3-butadiynes, one of the simplest method for the preparation of 1,3-butadiyne is copper catalyzed oxidative homo-coupling of terminal alkynes [16].

The cycloaddition reactions of alkynes is one of the most general methods for the construction of arenes and heterocyclic rings, and some review papers have summarized the advance of this research field [17,18,19]. The applications of 1,3-butadiynes as starting materials in cycloaddition/cyclo- condensation reactions show significantly different reactivity compared to monoalkynes to construct complex molecules bearing many more functional groups. This review presents an overview of the recent advances on the cycloaddition/cyclocondensation reactions of 1,3-butadiynes providing access to carbocycles and heterocycles.

## 2. Formation of Carbocycles

**Scheme 1:**
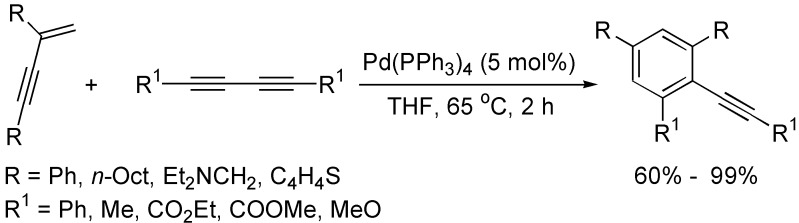
Synthesis of substituted benzene from enynes and 1,3-diynes.

The cycloaddition of 1,3-butadiynes with other unsaturated reactants is one of the most general methods for the construction of substituted benzenes. Gevorgyan and co-workers reported an efficient method for the synthesis of alkynyl-substituted benzenes by substituted enynes with 1,3-butadiynes at 65 °C in THF in the presence of Pd(PPh_3_)_4_, and the yields of benzene derivatives generally depended on the substituents, as well as the regioisomers were normally obtained in the case of unsymmetrical enynes used (Scheme 1) [20,21,22].

**Scheme 2:**
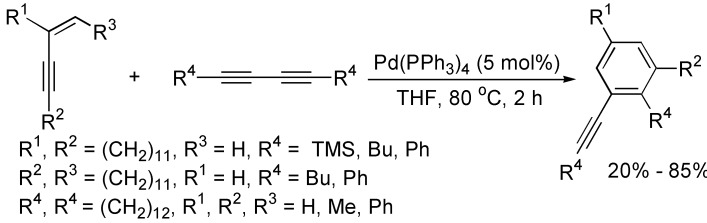
Synthesis of cyclophanes.

The same group described a standard method for preparation of several different types of cyclophanes via an intermolecular palladium-catalyzed cross-benzannulation reaction of cyclic enynes with 1,3-diynes or enynes with cyclic 1,3-diynes (Scheme 2) [23].

**Scheme 3:**
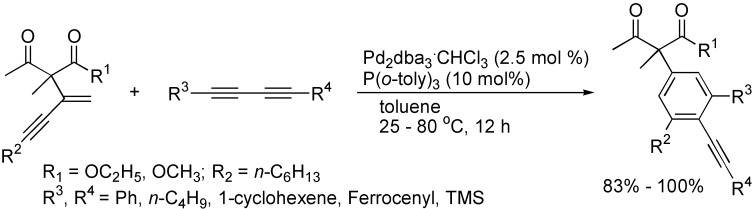
Synthesis of 3-oxobutanoates.

α-Arylation of carbonyl compounds is an intrinsically more difficult reaction, which creates problems for synthesis chemists. Nakamura and co-workers introduced a novel and general method for the synthesis of functionalized α-aryl carbonyl compounds by the [4+2] benzannulation of β-keto esters with 1,3-diynes (1.2 equiv) in the presence of Pd(0) catalyst, providing an effective route for the synthesis of arylated 3-oxobutanoates (Scheme 3) [24].

**Scheme 4:**
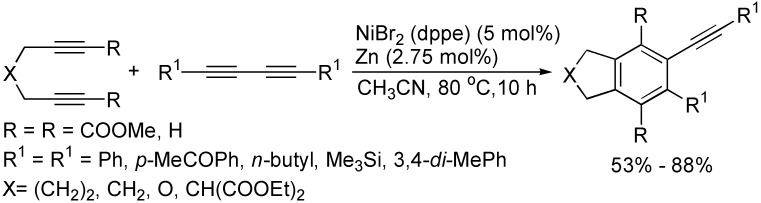
Synthesis of alkynyl benzene derivatives.

In the presence of NiBr_2_ and zinc powder, the chemo- and regioselectively controlled [2+2+2] cycloaddition of nonconjugated diynes with 1,3-butadiynes at 80 °C in CH_3_CN afforded the potential alkynyl benzene synthetic intermediates in 53%–88% yields. (Scheme 4) [25].

**Scheme 5:**
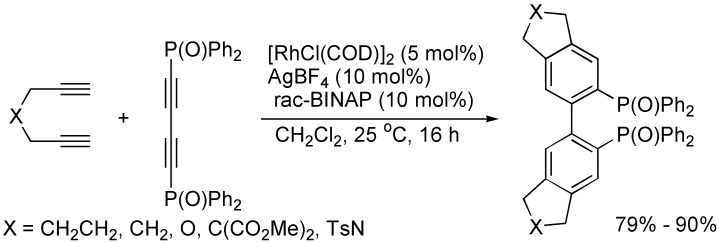
Synthesis of biaryl diphosphines.

Rhodium/silver-catalyzed double [2+2+2] cycloaddition of 1,4-bis(diphenylphosphinoyl)-1,3-butadiyne with tethered diynes, which are nonconjugated diynes at room temperature, provided a straightforward, one-pot procedure for the synthesis of biaryl diphosphines with a variety of substitution patterns and functionalities (Scheme 5) [26].

**Scheme 6:**
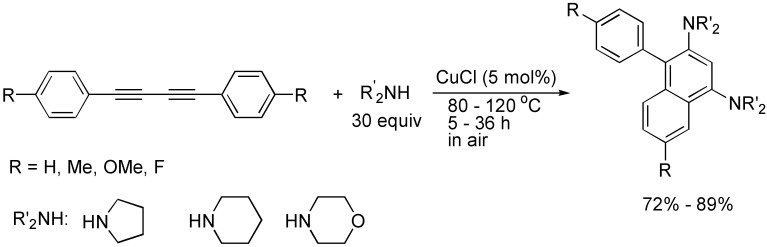
Synthesis of aminonaphthalene derivatives.

The cycloaddition reactions of 1,3-butadiynes have been also demonstrated to be the efficient reactions for the construction of naphthalene rings. For example, in the presence of CuCl, 1,4-diaryl-1,3-butadiynes reacted with cyclic amines such as pyrrolidine, piperidine and morpholine to afford amino-substituted naphthalene derivatives in good to high yields. The catalytic procedure provided an atom-economic, one-pot synthetic method for the synthesis of functionalized naphthalene derivatives, which are not easily prepared by traditional known organic transformations (Scheme 6) [27].

**Scheme 7:**
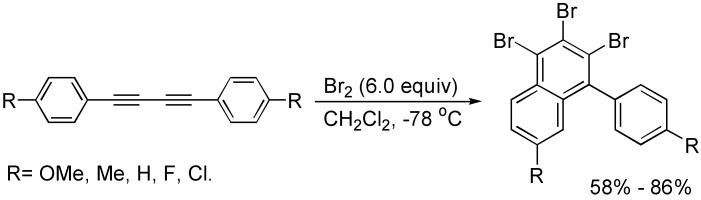
Synthesis of bromonaphthalene.

In the presence of bromine, 1,4-diaryl-1,3-butadiynes with bromine underwent an intramolecular cyclization at low temperature to afford 1,2,3-tribromonaphthalenes, which are very useful intermediates for the synthesis of other functionalized naphthalenes via the cross-coupling reaction of carbon-bromide bonds (Scheme 7) [28].

## 3. Formation of Heterocycles

The cycloaddition reactions of 1,3-butadiynes with other reactants having heteroatoms is one of the most interesting and intriguing subjects in the synthesis of heterocyles. As a result of these reactions, very important and useful heterocyles such as oxygen-, nitrogen-, sulfur-, silicon-, and metal-containing cyclic compounds can be easily obtained.

### 3.1. Oxygen-Containing Heterocycles

**Scheme 8:**
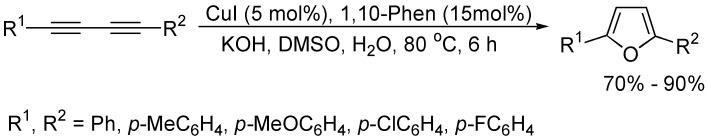
Synthesis of 2,5-disubstituted furans.

**Scheme 9:**
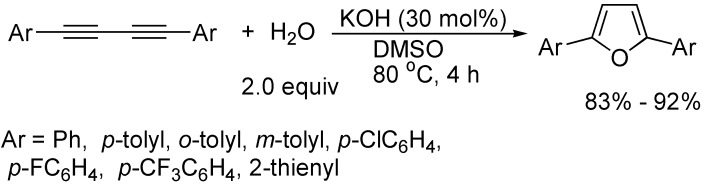
Synthesis of 2,5-diarylfurans.

The cycloaddition of 1,3-butadiynes with water has been developed as an efficient and simple way to synthesize 2,5-disubstituted furan derivatives. For example, in the presence of CuI and 1,10-phenanthroline, and using KOH as a base in DMSO, 2,5-diarylfurans could be obtained in good yields (Scheme 8) [29]. The same cycloaddition reaction also occurred catalyzed by Pd(PPh_3_)_4_/2,5-norbornadiene with the use of KOH as a base [30]. Very interestingly, after optimizing the reaction conditions, it was found that in DMSO, only KOH could efficiently catalyze the cycloaddition of 1,4-diaryl-1,3-butadiynes with water to afford 2,5-diarylfurans in good to high yields (Scheme 9) [31].

**Scheme 10:**
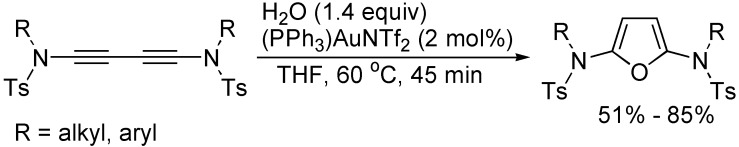
Synthesis of 2,5-diaminofurans.

In addition, Au(I)-catalyzed hydration of 1,4-diamino-1,3-butadiynes led to preparation of 2,5-diaminofurans in good yields under mild conditions, and tolerated various functional groups (Scheme 10) [32].

**Scheme 11:**
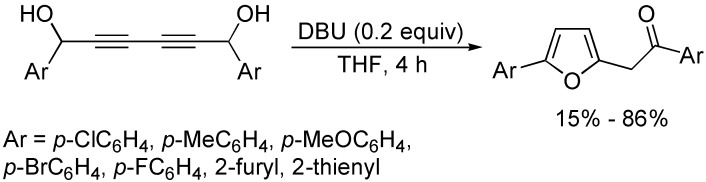
Synthesis of 2,5-disubstituted furan derivatives.

The intramolecular hydroalkoxylation of alkynes has been deeply studied to provide a straightforward methodology for the construction of oxygen-containing heterocycles. In the presence of DBU, the intramolecular cyclization reaction of diynyl-1,6-diols involving a cascade 1,3-H shift and Michael addition to construct furan derivatives (Scheme 11) [33]. It was suggested that 1,3-H shift was the rate-determining step, and the regioselectivity depended on the substituents attached to the aryl groups.

**Scheme 12:**
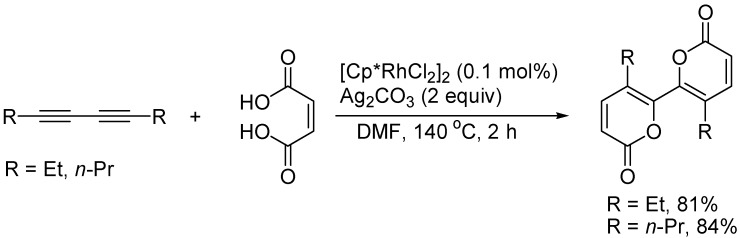
Synthesis of α-pyrone derivatives.

α-Pyrones with various substituents have been found in a wide range of natural products with interesting biological and physiological activity. As shown in Scheme 12, bis-(α-pyrone) could be synthesized from the rhodium-catalyzed reaction of 1,3-butadiynes with maleic acids accompanied by decarboxylative and dehydrogenative cyclocondensation (Scheme 12) [34].

**Scheme 13:**
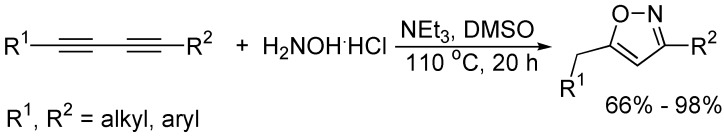
Synthesis of disubstituted isoxazoles.

The cycloaddition of 1,3-butadiynes with hydroxylamine hydrochloride (2.5 equiv) in the presence of triethylamine in DMSO afforded 3,5-disubstituted isoxazoles in satisfactory to excellent yields, in which the intermolecular Cope-type hydroamination of 1,3-butadiynes with NH_2_OH^.^HCl and the intramolecular electrophilic addition are involved (Scheme 13) [35].

### 3.2. Nitrogen-Containing Heterocycles

**Scheme 14:**
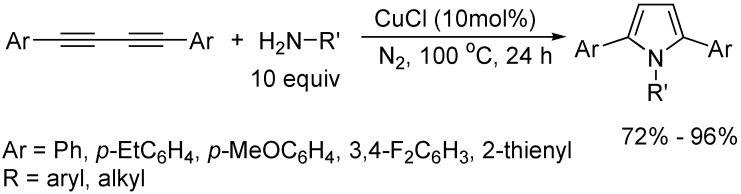
Synthesis of 1,2,5-trisubsituted pyrroles.

**Scheme 15:**
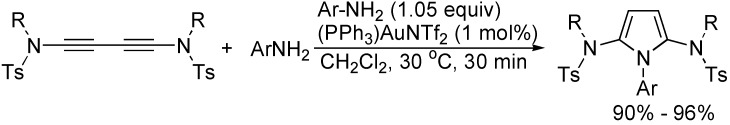
Synthesis of pyrrole derivatives.

The cycloaddition of 1,3-butadiynes with N-H bond is an important way to construct nitrogen-containing heterocycles, when 1,3-butadiynes underwent inter- and intramolecular double hydroamination with primary amines (10 equiv) in the presence of CuCl, 1,2,5-trisubsituted pyrroles could be prepared in good to high yields, providing an atom-economic process for the synthesis of pyrrole derivatives (Scheme 14) [36]. (PPh_3_)AuNTf_2_ could also catalyze a similar cycloaddition reaction to give 2,5-diaminopyrroles in high yields (Scheme 15) [32].

**Scheme 16:**
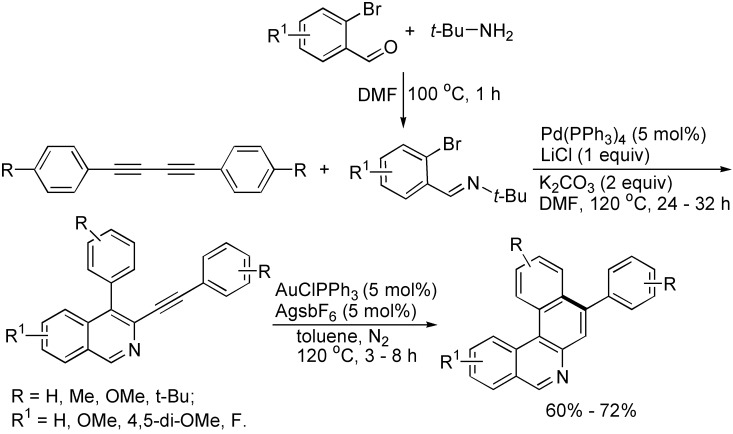
Synthesis of isoquinoline derivatives.

On the basis of the cycloaddition reactions of two carbon-carbon triple bonds, 1,3-butadiynes have been one of the simple and efficient starting materials for the formation of polyaromatic compounds. For example, 8-aryl-substituted benzo[a]phenanthridine derivatives could be obtained by consecutive three component tandem reactions of 2-bromobenzaldehydes, *t*-butylamine and 1,3-butadiynes involving palladium-catalyzed cyclocondensation of 1,3-butadiynes with (2-bromobenzylidene)-*t*-butylamine generated *in situ* giving 3-alkynyl isoquinoline intermediates, which then underwent 6-endo carbocyclization in the presence of AuCl(PPh_3_)/AgSbF_6_ (Scheme 16) [37].

**Scheme 17:**
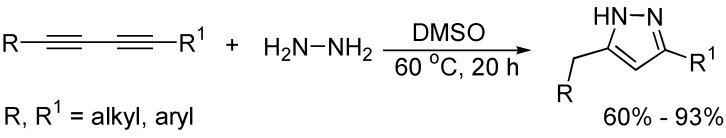
Synthesis of 3,5-disubstituted pyrazoles.

3,5-Disubstituted pyrazoles could be also synthesized via Cope-type hydroamination of 1,3-butadiynes with aqueous hydrazine solution in DMSO as shown in Scheme 17. The cycloaddition reaction occurred smoothly to give the products in satisfactory to excellent yields without use of catalyst, providing a practical and useful access to pyrazole derivatives [38].

**Scheme 18:**
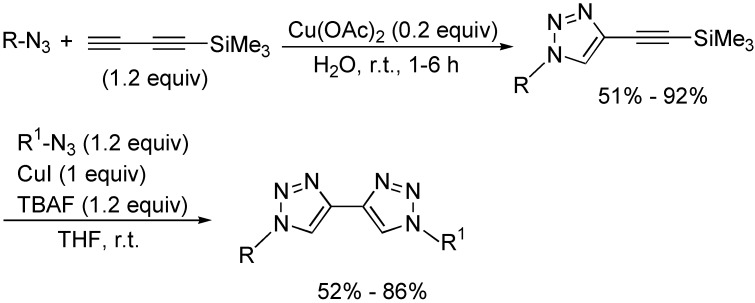
Synthesis of 4,4'-bi-1,2,3-triazole derivatives.

1,2,3-Triazole derivatives have found a wide range of important applications in the agrochemical, pharmaceutical, and organic material fields [39,40]. They are conveniently synthesized from the cycloaddition of alkynes with azides. Recently, several cycloaddition reactions of 1,3-butadiynes with azides have been developed to provide interesting and useful synthetic methods to construct novel 1,2,3-triazole derivatives. As shown in Scheme 18, unsymmetrically substituted 4,4'-bi-1,2,3-triazole derivatives could be prepared from the reaction of the easily available 1-trimethylsilyl-1,3-butadiyne with two kinds of different azides in two steps in the presence of copper salts as catalysts. In addition, tetrabutylammonium fluoride (TBAF) was used as the desilylating agent in this transformation [41].

**Scheme 19:**
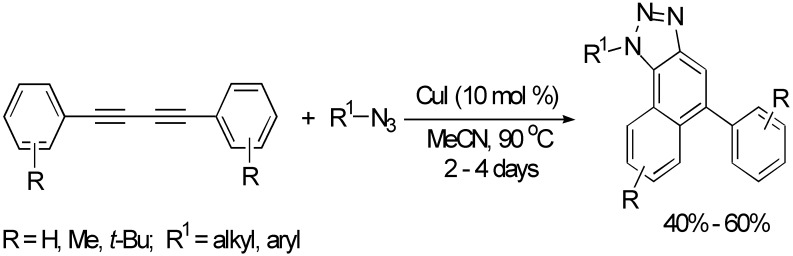
Synthesis of naphthotriazoles.

Naphthotriazoles could be obtained in moderate yields from the CuI-catalyzed one-pot sequential cyclization of 1,3-butadiynes with azides in MeCN at 90 °C for 2–4 days (Scheme 19) [42]. The protocol involves a sequential 1,3-dipolar cycloaddition and carbocyclization. 

**Scheme 20:**
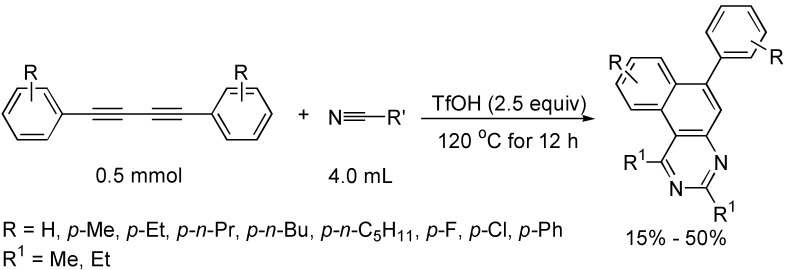
Synthesis of benzo[*f*]quinazoline derivatives.

A novel [4+2+2] cycloaddition of aryl-substituted 1,3-butadiynes with acetonitrile or propionitrile in the presence of trifluoromethanesulfonic acid afforded benzo[*f*]quinazolines with the considerable advantages of high atom-efficiency, one-pot operation and easy availability of the starting materials, although the yields of products were modest (Scheme 20) [43].

**Scheme 21:**
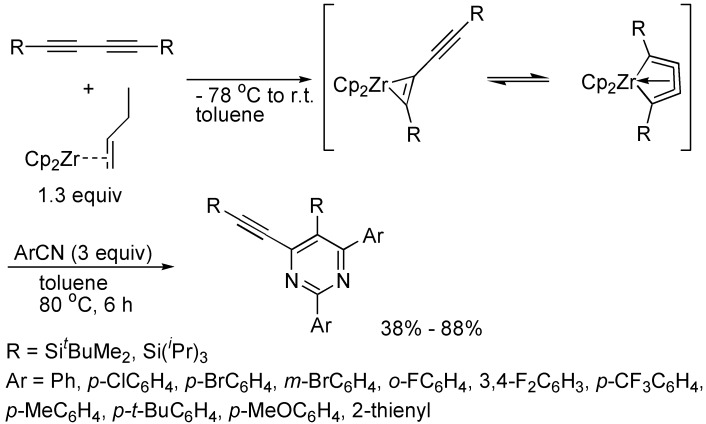
Synthesis of functionalized pyrimidines.

At low temperature, 1,3-butadiynes bearing bulky silyl groups reacted with the Negishi reagent resulting in the formation of five-membered zirconacyclocumulene intermediates, which reacted with two molecules of aryl nitrile at 80 °C affording with excellent regioselectivity polysubstituted pyrimidines including alkynyl groups. The electronic nature of the aryl nitriles had little influence on the yields, but the steric hindrance of aryl nitriles affected the yields greatly. Therefore, in the case of 2-fluorobenzonitrile employed, only 38% yield of the expected product was obtained (Scheme 21) [44].

### 3.3. Other Heterocycles

**Scheme 22:**
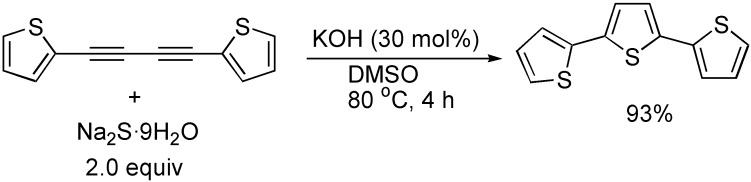
Synthesis of 2,2':5',2''-terthiophene.

**Scheme 23:**
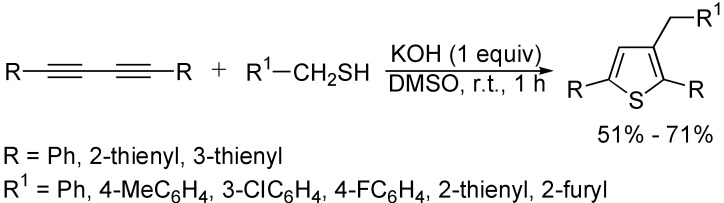
Synthesis of thiophene derivatives.

As described above, the cycloaddition of 1,3-butadiynes with hydrogen-heteroatom bonds is one of the more efficient and simple reactions for synthesizing five-membered furan and pyrrole heterocycles, and there have been some reports on the synthesis of 2,5-disubstituted thiophenes from the reaction of 1,3-butadiynes with Na_2_S**^.^**9H_2_O in the presence of catalyst [29], or without catalyst [45]. Very recently, the synthesis of 2,2’:5’,2”-terthiophene, which is an interesting class of organic molecules with interesting electrical and/or optical properties, via the cyclocondensation reaction of 1,4-bis(2-thienyl)-1,3-butadiyne with Na_2_S**^.^**9H_2_O in the presence of KOH in DMSO was reported (Scheme 22) [31]. 2,3,5-Trisubstituted thiophene derivatives could be also obtained from the reaction of 1,4-diaryl-1,3-butadiynes with one equivalent of arylmethanethiol in DMSO promoted by KOH (Scheme 23) [46].

**Scheme 24:**
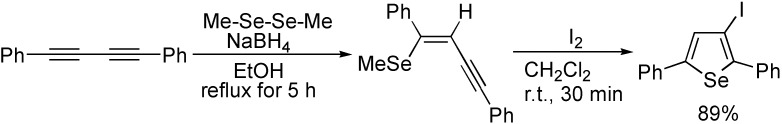
Synthesis of substituted selenophenes.

Treatment of 1,4-diphenyl-1,3-butadiyne with methaneselenolate anion, generated *in situ* from dimethyldiselenide and NaBH_4_ in ethanol under reflux produced the corresponding (*Z*)-selenoenyne in 70% yield, which underwent a cyclization reaction with I_2_ at room temperature to afford 2,5-diphenyl-3-iodoselenophene in 89% yield (Scheme 24) [47].

**Scheme 25:**
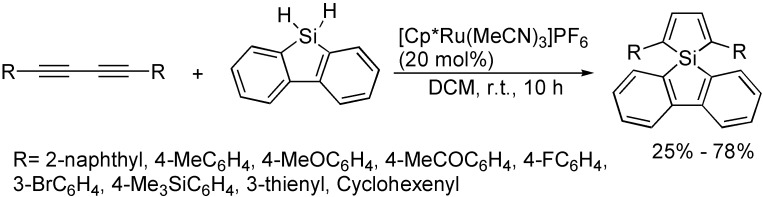
Synthesis of *spiro*-type 2,5-diarylsiloles.

As shown in Scheme 25, in the presence of [Cp*Ru(MeCN)_3_]PF_6_, the double hydrosilylation of 1,3-butadiynes with 3.0 equivalents of 9-silafluorene provided a novel synthetic route for *spiro*-type 2,5-diarylsiloles under mild reaction conditions [48]. The cyclohydrosilylation greatly depended on the structures of dihydrosilanes, Ph_2_SiH_2_ and PhSiClH_2_ showed mild reactivity, but Et_2_SiH_2_ and PhMeSiH_2_ showed no reactivity.

## 4. Formation of Metallacyclopentadienes

Metallacyclopentadienes are metal-containing heterocycles, which are considered to be the key intermediates for the formation of aromatic derivatives, particularly in the construction of benzene derivatives from the trimerization of alkynes in the stoichiometric and catalytic reactions [49,50]. The reactions of transition-metal complexes with 1,3-butadiynes provide an important and useful ways approach such type of metal-containing heterocycles.

**Scheme 26:**
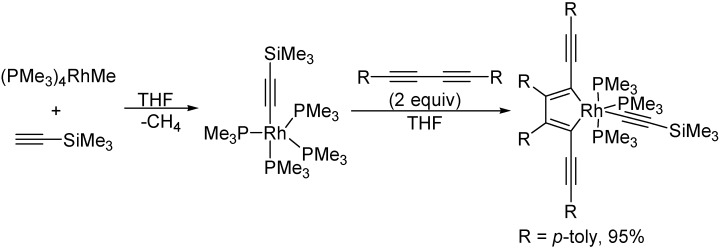
Synthesis of rhodacyclopenta-2,4-diene complex.

Rourke and co-workers reported the preparation of 2,5-bis(arylethynyl)metallacyclopentadiene in high yield via the sequential reaction of [(PMe_3_)_4_RhMe] with one equivalent of ethynyltrimethylsilane, and then with two equivalents of 1,4-diaryl-1,3-butadiynes at room temperature in THF (Scheme 26) [51].

**Scheme 27:**
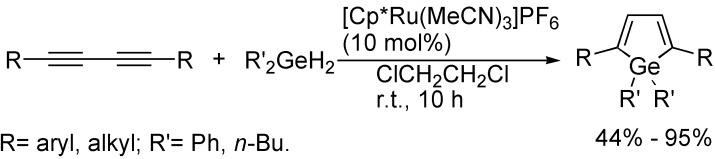
Synthesis of germole derivatives.

The formation of metallacyclopentadienes could also be achieved from the simple cycloaddition reaction of 1,3-butadiynes with metal-hydrogen bonds. For example, in the presence of [Cp*Ru(MeCN)_3_]PF_6_, the double *trans*-hydrogermylation of 1,3-butadiynes with 1.5 equivalents of dihydrogermane in 1,2-dichloroethane at room temperature afforded 2,5-disubstituted germoles in good to high yields (Scheme 27) [52].

## 5. Conclusions

In this review, we have presented an overview of the advances on the cycloaddition/condensation of 1,3-butadiynes to construct a variety of functionalized carbo- and heterocycles with the advantages of atom-efficiency and one-pot reaction. The reactions shown in this review have demonstrated the versatile reactivity and important applications of 1,3-butadiynes in the synthesis of diverse ring systems. It is reasonable to assume that development of the new reactions of 1,3-butadiynes will continue to be a challenging research topic in organic synthesis.
